# Disruption of the Axonal Trafficking of Tyrosine Hydroxylase mRNA Impairs Catecholamine Biosynthesis in the Axons of Sympathetic Neurons

**DOI:** 10.1523/ENEURO.0385-16.2017

**Published:** 2017-06-16

**Authors:** Armaz Aschrafi, Anthony E. Gioio, Lijin Dong, Barry B. Kaplan

**Affiliations:** 1 Laboratory of Molecular Biology Division of Intramural Research Programs, National Institute of Mental Health National Institutes of Health, Bethesda, MD 20892; 2 Genetic Engineering Core, National Eye Institute National Institutes of Health, Bethesda, MD 20892

**Keywords:** 3’untranslated region, CRISPR Cas9, dopamine, local translation, mRNA trafficking, superior cervical ganglion

## Abstract

Tyrosine hydroxylase (TH) is the enzyme that catalyzes the rate-limiting step in the biosynthesis of the catecholamine neurotransmitters. In a previous communication, evidence was provided that TH mRNA is trafficked to the axon, where it is locally translated. In addition, a 50-bp sequence element in the 3′untranslated region (3’UTR) of TH mRNA was identified that directs TH mRNA to distal axons (i.e., zip-code). In the present study, the hypothesis was tested that local translation of TH plays an important role in the biosynthesis of the catecholamine neurotransmitters in the axon and/or presynaptic nerve terminal. Toward this end, a targeted deletion of the axonal transport sequence element was developed, using the lentiviral delivery of the CRISPR/Cas9 system, and two guide RNA (gRNA) sequences flanking the 50-bp *cis-*acting regulatory element in rat superior cervical ganglion (SCG) neurons. Deletion of the axonal transport element reduced TH mRNA levels in the distal axons and reduced the axonal protein levels of TH and TH activity as measured by phosphorylation of SER40 in SCG neurons. Moreover, deletion of the zip-code diminished the axonal levels of dopamine (DA) and norepinephrine (NE). Conversely, the local translation of exogenous TH mRNA in the distal axon enhanced TH levels and activity, and elevated axonal NE levels. Taken together, these results provide direct evidence to support the hypothesis that TH mRNA trafficking and local synthesis of TH play an important role in the synthesis of catecholamines in the axon and presynaptic terminal.

## Significance Statement

Tyrosine hydroxylase (TH) is the rate-limiting enzyme in the biosynthesis of the catecholamine neurotransmitters. Previous results suggested that TH mRNA is trafficked to the distal axons of primary sympathetic neurons and is locally translated. In the present study a gene editing strategy was employed to delete the axonal TH mRNA trafficking regulatory element (i.e., zip-code). Deletion of the zip-code reduced TH mRNA levels in the distal axons, reduced axonal protein levels of TH and diminished the axonal levels, and release of norepinephrine (NE). Collectively, these studies demonstrate that the local synthesis of TH plays an important role in catecholamine synthesis and may facilitate the maintenance of catecholamine levels in response to long-term alteration in the need for neurotransmitters.

## Introduction

It is now evident that one of the mechanisms used by axons and presynaptic nerve terminals to rapidly respond to extracellular cues is to temporally and spatially regulate protein expression by asymmetrical mRNA transport and localized translation (Gomes et al., 2014; Jung et al., 2014). Transport of mRNAs into the distal axons enables coordination of local changes in the presynaptic proteome, a process critical in directing the growth cones of immature axons and ensuring neuronal survival (Fallini et al., 2016). These axonal mRNAs code for several functional categories of proteins including: cytoskeletal elements, ribosomal proteins and translation factors, transcription factors, as well as metabolic enzymes and nuclear-encoded mitochondrial mRNAs (Scott et al., 2015). The targeting of specific mRNAs to the distal structural/functional domains of the neuron is driven by sequence motifs located in the 3′untranslated region (3’UTR) of the RNA itself, which are recognized by specialized RNA-binding proteins (RBPs; Smart et al., 2007). These *cis*-acting regulatory elements or “zip-codes” contain localization signals (Aschrafi et al., 2010). The motifs can be constituted by either the primary sequence of the mRNA, or its higher order structure, such as a hairpin or stem-loop (Aschrafi et al., 2010; Di Liegro et al., 2014).

In addition to mRNAs coding for structural, mitochondrial, ribosomal proteins, and translation regulatory elements, there is considerable evidence to suggest that mRNAs encoding proteins involved in neurotransmitter synthesis are present in the axon. Recent research suggested that tyrosine hydroxylase (TH) mRNA is localized to axons of sympathetic neurons, and demonstrated that its local translation facilitates axonal dopamine (DA) synthesis (Gervasi et al., 2016). The finding that TH mRNA is present in axons of primary sympathetic neurons is consistent with the detection of TH mRNA in brain regions innervated by catecholaminergic terminals (Lewis et al., 1991; Melia et al., 1994). In the adult rat brain, the presence of TH mRNA transcripts was reported in the cerebellum, striatum, and pituitary neurointermediate lobe, all regions that are devoid of catecholamine-synthesizing cells, but receive significant catecholaminergic innervation (Melia et al., 1994). Moreover, a 50-bp sequence element in the 3’UTR of TH mRNA that directs TH mRNA to distal axons has been identified (Gervasi et al., 2016). This TH mRNA zip-code was also shown to be sufficient to mediate the trafficking of a reporter mRNA to distal axons of sympathetic neurons.

Traditional approaches to studying the functionality of *cis*-acting regulatory sequences that control cellular mRNA trafficking *in vitro* involve overexpression of sequences linked to a reporter gene (Kaplan et al., 2009). These experiments are hampered by the out-of-genomic-context, in which the noncoding elements are studied, as well as untoward consequences of introducing abnormally high levels of foreign DNA sequences that could perturb endogenous gene expression and basic neuronal function (Han et al., 2015). The CRISPR/Cas9 system has recently been harnessed as an RNA-guided nuclease method of genome editing *in vitro* and *in vivo* in several animal species (Hsu et al., 2014). While this programmable endonuclease approach to genome editing has been deployed numerous times to modify protein-coding genes, there has yet to be a report on its use in altering the regulatory elements controlling mRNA trafficking. Here, we employed the CRISPR-Cas9 system to test the hypothesis that the axonal trafficking of TH mRNA and its local translation plays an important role in the biosynthesis of the catecholamine neurotransmitters in the axon and/or presynaptic nerve terminal. The outcome of our studies clearly demonstrate that CRISPR-mediated genomic deletion of the axonal transport element of TH mRNA abolishes axonal trafficking of TH mRNA and diminishes presynaptic catecholamine synthesis and release in primary rat sympathetic neurons.

## Materials and Methods

### Neuronal cell cultures

Superior cervical ganglia (SCGs) were removed from 3-d-old Harlan Sprague Dawley rats of either sex and dissociated to single cell suspensions using gentleMACS Dissociator and Neuronal Tissue Dissociation kit (Miltenyi Biotec). The dissociated cells were plated in the center compartment of three-chamber Campenot culture dishes (Hillefors et al., 2007) or, for purpose of *in situ* hybridization histochemistry, onto Nunc Lab-Tek II-CC2 glass chamber slides (Sigma-Aldrich). Cells were cultured in serum-free Neurobasal medium (ThemoFisher) supplemented with 2% B_27_ supplement, 0.5 mM L-glutamine (ThemoFisher), 25 μM glutamic acid (Sigma-Aldrich), 50 ng/ml NGF, 20 mM KCl, 20 U/ml penicillin and 20 µg/ml streptomycin (Hyclone) for 3–14 d *in vitro* (DIV) before use. Medium was changed every 3–4 d. Two days after plating, 5-fluoro-2’-deoxyuridine (50 µM) was added to the culture medium to inhibit growth of non-neuronal cells. After 7–14 DIV, distal axons were harvested from the side compartments of the culture chambers, which were devoid of neuronal soma or non-neuronal cells, as judged by phase-contrast microscopy.

### CRISPR design and synthesis

**Figure 1.**
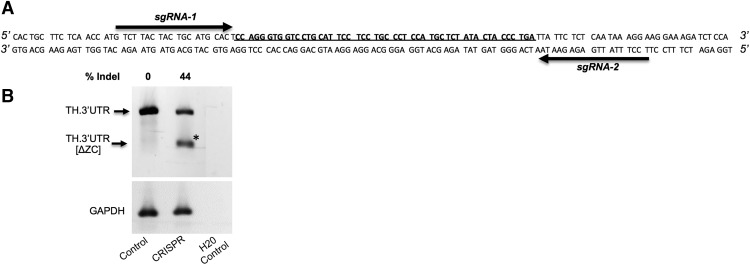
Axonal targeting sequence present in the rat TH mRNA can be removed by a lentiviral vector containing CRISPR/Cas9. ***A***, LentiCRISPR/Cas9-mediated deletion of the axonal targeting element (i.e., zip-code) of TH mRNA. Location of sgRNAs sequences targeting the flanking ends of the 50-bp region containing the axonal targeting sequence in the 3’UTR of rat TH mRNA. Diagram shows the gRNA-targeting sites. The 50-bp zip-code sequence is bolded. ***B***, PCR analysis of genomic DNA extracted from SCG neurons infected with lentiCRISPR (control) or sgRNA 1 and sgRNA 2 lentiCRISPR (CRISPR) virus. One week after infection, genomic DNA from the cells was extracted and amplified using PCR to test for the deletion of the 50-bp region. The faster migrating band (indicated by arrow) is the shortened TH allele. GAPDH was used as a loading control. The percentage of edited DNA that resulted in an indel (% indel) was determined by quantitating the amount of uncut versus cut DNA (*).

To minimize off-target activity, the RNA-guided interspaced short palindromic repeats (CRISPR)–Cas9 nuclease system was combined with a double nicking strategy to introduce targeted DNA double-stranded breaks around the zip-code region. DNA targeting was performed using two CRISPR guide RNAs (gRNAs), which bound flanking regions of the 50-bp zip-code located in the 3’UTR of the TH gene (Fig. 1*A*). gRNAs were designed using an online CRISPR Design Tool (crispr.mit.edu), and generated by the slow annealing and phosphorylation of two complementary oligonucleotides (IDT) with T4 polynucleotide kinase (New England Biolabs) in a 10 µL reaction mixture. gRNA1 forward oligo (Fw): 5’ CACCGTTACTACTGCATGCACTCCA 3’; reverse oligo (Rv): 5’ CAATGATGACGTACGTGAGGTCAAA 3’; gRNA2 Fw oligo: 5’ CACCGCCTTTATTGAGAGAATAATC 3’; Rv oligo: 5’ AAACGATTATTCTCTCAATAAAGGC 3’ (target sequence is underlined). The pLentiCRISPR v.2 vector (AddGene) was digested using BsmBI, and a 1:250 dilution of annealed oligos were cloned into the single gRNA scaffold of the linearized pLentiCRISPR vector backbone as described by (Sanjana et al., 2014). Plasmid purification and endotoxin removal for CRISPR constructs were performed using the GenElute HP Plasmid Maxiprep kit (Sigma-Aldrich). Correct orientation of the cloned gRNAs was confirmed using DNA Sanger sequencing. Cas9 cleavage efficiency was determined in primary SCG neurons 5–7 d after coinfection with the gRNA1 and gRNA2 containing lentiCRISPR. Genomic DNA was extracted and RNase A-treated using Gentra Puregene Cell kit (QIAGEN). Isolated genomic DNA was subjected to PCR amplification using primers that flanked the predicted Cas9 cut site regions, Fw: 5’ CTGGACAGCCCTCACACCA 3’, Rv: 5’ GAACTATCTTTGTGGTGACGCAG 3’. Amplicons from target cleavage sites were ligated into plasmids (JetClone kit, ThermoFisher) and transfected into high-efficiency DH5α competent *Escherichia coli* cells (ThermoFisher) for DNA Sanger sequencing analysis of 8–10 clones, from which the ratios of genomic deletion were used to quantify cleavage efficiency.

### Lentivirus production

Viral particles were prepared by cotransfection of HEK-293T cells with gRNA-cloned transfer lentiCRISPRv2 plasmids together with the packaging plasmids pVSVg (AddGene) and psPAX2 (AddGene) using Lipofectamine2000 (ThermoFisher). HEK293T cells were placed overnight in a 5% CO_2_ humidified 37°C incubator. The following day, medium was replaced by fresh Ultraculture (Lonza) and supernatants were collected 24 h later. Viral particles were purified using a Lenti-X Maxi Purification kit (Takara) and stored at −80°C until use.

### Constructs and SCG neuron transfection

Full-length or zip-code-truncated TH mRNAs were synthesized as described by Gervasi et al. (2016). DNA oligonucleotides encoding the full-length rat TH ORF with the entire 3’UTR, or 3’UTR lacking the 50-bp zip-code sequence (IDT) were cloned after the T7-promoter of the pBLUE vector using the ECORI and HindIII restriction sites. Following vector linearization, using the HindIII site, 5′ methyl cap containing TH mRNA was synthesized using the mMESSAGE mMACHINE T7 Transcription kit (Ambion). Purified TH mRNA (0.5 μg) was transfected into distal SCG axons present in the lateral compartment of Campenot chambers using mRNA-In Neuro (GlobalStem).

### RNA isolation and quantitative reverse transcriptase PCR (qRT-PCR)

RNA was isolated using Direct-Zol RNA MiniPrep (Zymo Reasearch) according to the manufacturer’s instructions. Briefly, cells and proximal axons cultured in the central compartment of Campenot chambers or distal axons growing in the side compartments were lysed in 100 μl of TRIzol (ThermoFisher). An equal volume of ethanol was added to the homogenate and the mix was loaded onto a Zymo-Spin IIC Column and washed with Direct-Zol RNA PreWash and Wash buffers. The RNA sample was eluted in 35 μl of water. On average, the concentration of the purified RNA was 15 ng/μl for RNA isolated from distal axons and 25 ng/μl for RNA isolated from neuronal cell bodies and proximal axons. Reverse transcription was performed using RevertAid H Minus First Strand cDNA Synthesis kit (ThermoFisher). For qRT-PCR analysis, 2 μl of 1:10 diluted cDNA was added to 10 μl Fast SYBR green (Affymetrix) and 2 μl of QuantiTect primers (QIAGEN). For each experimental sample, the reaction was run in triplicate, using a StepOne Real-Time PCR system (ThermoFisher). The results were analyzed using the StepOne Software (ThermoFisher), normalizing target mRNA levels to β-actin mRNA levels. TH, GAPDH, and β-actin gene primers were QuantiTect validated primers (QIAGEN). The following primers we used For RT-PCR analysis of intact and zip-code-less TH mRNA: Fw: 5’ ATCCAGCGCTCCTTGG, Rv: 5’ AGATTCTTTCCTTCCTTTATTGAGA.

### *In situ* hybridization histochemistry

*In situ* hybridization was performed on freshly fixed tissue using the RNAscope 2.5 High Definition-RED Assay (Advanced Cell Diagnostics) according to manufacturer’s instructions. Cultured neurons were fixed in 20% paraformaldehyde (PFA) for 30 min, dehydrated in increasing concentrations of ethanol (50%, 70%, and 100%) and rehydrated (70% ethanol, 50% ethanol, PBS) before applying Pretreat 1 and 1:15 diluted pretreat three reagents. A target specific riboprobe (rat TH, RefSeq: NM_012740.3) was then hybridized overnight at 40°C, followed by incubation with signal amplification reagents. The hybridized riboprobe was visualized with RED2.5 under either bright field or fluorescence microscopy using a Texas Red filter.

### Immunocytochemistry

Neurons were fixed using 10% PFA for 30 min. After washing, permeabilization was accomplished by incubation with 0.3% Triton X-100 in PBS for 3 min. Neurons were then blocked in 5% bovine serum albumin (BSA) in PBS for 30 min at room temperature (RT) and incubated in primary antibody diluted in 3% BSA-PBS for 4 h at RT or overnight at 4°C. After incubation with the primary antibodies [anti-TH antibody, Cell Signaling, 1:1000; anti-phospho-TH (Ser40) antibody, Cell Signaling 2791S, 1:500; anti-β-actin antibody, Cell Signaling, 1:1000; anti-dopamine (DA) antibody, Abcam, 1:1000; anti-norepinephrine (NE) antibody, Abcam, 1:1000], neurons were washed in 0.1% Triton X-100 PBS, incubated in secondary antibody [Alexa Fluor 594 (or 488) goat anti-mouse or goat anti-rabbit] for 1 h at RT and visualized using an EVOS fluorescence microscope.

### Western blot analysis

Axon or soma were lysed in RIPA buffer (Sigma-Aldrich) containing anti-peptidase cocktail (Roche). Lysates were heated in LDS sample buffer and 1 mM dithiothreitol, and separated on NuPAGE Novex 4–12% Bis-Tris Gels (ThermoFisher). Proteins were transferred to a Odyssey nitrocellulose membrane (LI-COR Biosciences), and membranes were blocked with Odyssey blocking buffer (LI-COR Biosciences) for 1 h. Proteins were detected by overnight incubation at 4°C with antibodies against TH (Cell Signaling, dilution 1:1000), anti-phospho-TH (Ser40) antibody (Cell Signaling, dilution 1:500), or β-actin (Cell Signaling, dilution 1:1000) in Odyssey blocking buffer containing 0.2% Tween 20 for either 2 h at RT or overnight at 4°C, followed by incubation with a IRDye 800CW-conjugated goat anti-rabbit IgG (LI-COR Biosciences) at a 1:5000 dilution in Odyssey blocking buffer containing 0.2% Tween 20 and 0.1% SDS for 1 h at RT. The membrane was washed 3 times with Tris-buffered saline/0.1% Tween 20 (TBS–T) between each incubation step. The membrane was then imaged with the Odyssey Infrared Imaging System (LI-COR Biosciences).

### Measurement of NE release

Primary SCG neurons grown in Campenot chambers (DIV 14) were infected with the empty lentiCRSPR or the gRNA-containing lentiCRISPR. One week after infection, cells were depolarized by the addition of 100 mM KCl into the distal axon-containing lateral compartment of Campenot chambers for 10 min. Culture media was collected and NE was measured using the Norepinephrine Research ELISA kit (Rocky Mountain) in accordance with the manufacturer’s instructions. Briefly, NE is bound to the solid phase of the microtiter plate. Acylated NE from the sample and solid phase-bound noradrenaline compete for a fixed number of antiserum binding sites. When the system is in equilibrium, free antigen and free antigen-antiserum complexes are removed by washing. The antibody bound to the solid phase NE was detected by anti-rabbit IgG/peroxidase. The substrate TMB/peroxidase reaction was monitored at 450 nm. The amount of antibody bound to the solid phase NE is inversely proportional to the NE concentration of the sample. Absorbance data were analyzed by linear regression analysis to calculate release NE levels.

### Bioinformatics and statistical analysis

The quantitative analyses of Western blot protein bands and cellular fluorescence signals, following reporter transfection and immunocytochemistry, was performed using ImageJ software as described previously (Burgess et al., 2010; McCloy et al., 2014). Quantitative data are presented as the mean ± SEM. Student’s *t* test or one-way ANOVA with Bonferroni multiple comparison was used for two- or three sample comparisons, respectively. Statistical significance was set at α = 0.05 for all experiments. Statistical analyses were performed using Microsoft Excel and GraphPad.

## Results

### CRISPR-mediated genome editing of axonal trafficking sequences located in the 3’UTR of TH mRNA

Previously, we employed chimeric fluorescent reporter constructs and identified a sequence element within the TH 3’UTR that is required for the axonal localization of the reporter mRNA (Gervasi et al., 2016). These results provided the first direct evidence that TH mRNA is trafficked to the axon under the direction of a *cis*-acting regulatory element situated in the 3’UTR of the mRNA. Results derived from cell fractionation experiments and PCR analyses established that TH mRNA was present in axonal polysomes. In addition, findings obtained from metabolic labeling studies demonstrated that the TH mRNA was locally translated. Based on these observations, it was hypothesized that the local translation of TH is critical for axonal TH activity and consequently the biosynthesis of the catecholamine neurotransmitters in the axon and/or presynaptic nerve terminal. To evaluate this postulate, we designed and tested paired RNA-guided CRISPR-associated nuclease Cas9 for introducing a targeted loss-of function deletion of the zip-code located in the 3’UTR of the TH gene. Lentiviral vectors were used for the delivery of two separate gRNAs directed to 5′ and 3′ ends of the zip-code (Fig. 1*A*). Infection of primary SCG neurons with Cas9 coupled gRNAs resulted in the deletion of the zip-code in 44% of the TH loci as detected by genomic PCR analyses (Fig. 1*B*).

### Deletion of the zip-code diminishes TH mRNA levels in the axon and reduces local TH synthesis and phosphorylation

**Figure 2.**
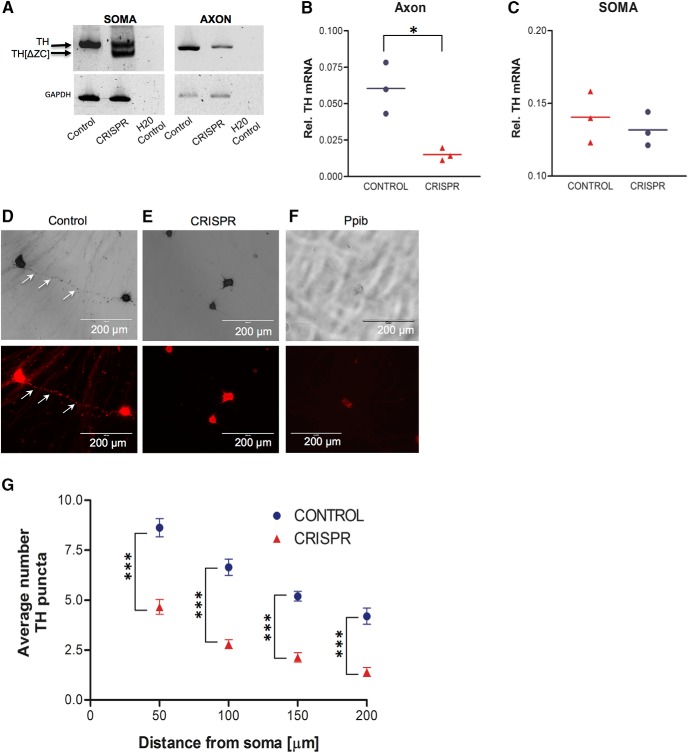
Deletion of the axonal transport element (zip-code) of TH mRNA significantly reduces TH mRNA levels in the axon. ***A***, RT-PCR analysis of intact and zip-code (ZC)-less TH mRNA in the axons and soma of SCG neurons one week after lentiCRISPR (control) or sgRNA 1 and sgRNA 2 lentiCRISPR (CRISPR) virus infection. Total RNA from the axons and soma of SCG neurons cultured in Campenot culture dishes was extracted, RNA was reverse transcribed into cDNA, and TH cDNA was amplified using PCR to examine the presence of intact and zip-code-less mRNA in the axons and soma of SCG neurons. GAPDH was used as a loading control. PCR amplification products were fractionated on a 4% agarose gel and visualized using an ultraviolet (UV) light. CRISPR-infected neurons produce both intact and zip-code-less TH mRNA, with zip-code-less TH mRNA detected only in the soma of SCG neurons. ***B***, ***C***, Quantification of TH mRNA levels in the distal axons and soma of SCG neurons infected with lentiCRISPR (control) or sgRNA 1 and sgRNA 2 containing lentiCRISPR (CRISPR). TH mRNA levels were determined by qRT-PCR one week after viral infection, using total RNA samples prepared from SCG axons and soma and gene-specific primers for TH. The relative levels of TH transcript were normalized to β-actin mRNA to provide an internal control for reverse transcription and axonal density. Data are the mean ± SEM (*n* = 3). *p ≤ 0.01. ***D***, ***E***, *In situ* hybridization analysis of single axons from dissociated primary SCG neurons grown in monolayer cell culture. In axons hybridized with a TH-specific riboprobe, TH mRNA appears as discrete puncta, whereas CRISPR-mediated deletion of the zip-code of TH mRNA abolishes TH puncta in the distal axons of primary SCG neurons. Arrows denote TH mRNA puncta in the axon. ***F***, mRNA for Ppib, an endoplasmic reticulum-associated protein, is not localized to the axons. ***G***, The number of TH mRNA containing granules is decreased in CRISPR neurons as compared with control neurons. Data are mean ± SEM from the measurement of 35–45 axons. TH mRNA containing puncta are measured as a function of axon length. The experiment was repeated three times with similar results. Student's *t* test, ***p ≤ 0.0001.

To assess whether the deletion of the zip-code affects axonal trafficking of TH mRNA, SCG neurons cultured in Campenot chambers were infected with viral particles encoding gRNAs/cas9 (CRISPR) or cas9 (control), and a RT-PCR analysis of the total RNA isolated from the axons and the infected somas was performed. Agarose gel fractionation of the RT-PCR products revealed that CRISPR-infected neurons produced both intact and zip-code-less TH mRNA (TH[ΔZC]), with the TH[ΔZC] mRNA detected solely in the soma of SCG neurons, whereas the full-length TH mRNA was PCR amplified from the soma and axonal RNA (Fig. 2*A*). The subsequent qRT-PCR analyses confirmed that, unlike the reduced TH mRNA quantities obtained in the distal axons of CRISPR-infected neurons (mean 0.06 ± 0.01 control vs 0.01 ± 0.002 CRISPR relative quantity, *p* ≤ 0.01; Fig. 2*B*), nearly equal amounts of the TH transcripts were present in the cell bodies 5 d after CRISPR infection (mean 0.14 ± 0.01 control vs 0.13 ± 0.01 CRISPR relative quantity, *p* ≤ 0.51; Fig. 2*C*), indicating that genomic deletion of the zip-code selectively reduces the abundance of axonal TH mRNA. These findings confirm previous results that the 50-bp zip-code is essential for the axonal localization of TH mRNA (Gervasi et al., 2016).

To confirm the absence of TH mRNA in axons of CRISPR-infected neurons, we used *in situ* hybridization histochemistry. In these experiments, the TH mRNA hybridization signal appeared as discrete puncta along the entire length of the axon bundles of control neurons (Fig. 2*D*). In contrast, no signal was detected in the axons of CRISPR-treated neurons, with most of the TH signal confined to the cell bodies (Fig. 2*E*). Measurement of the number of TH positive puncta as a function of axon length revealed that their number decreased by ∼50% in the axons of CRISPR-treated neurons as compared with control axons (Fig. 2*G*). In addition, the hybridization signal for the mRNA encoding an endoplasmic reticulum localized protein, peptidylprolyl isomerase B (Ppib), was restricted to the cell body, indicating that the TH signal detected in axons was due specifically to transport of TH mRNA (Fig. 2*F*).

**Figure 3.**
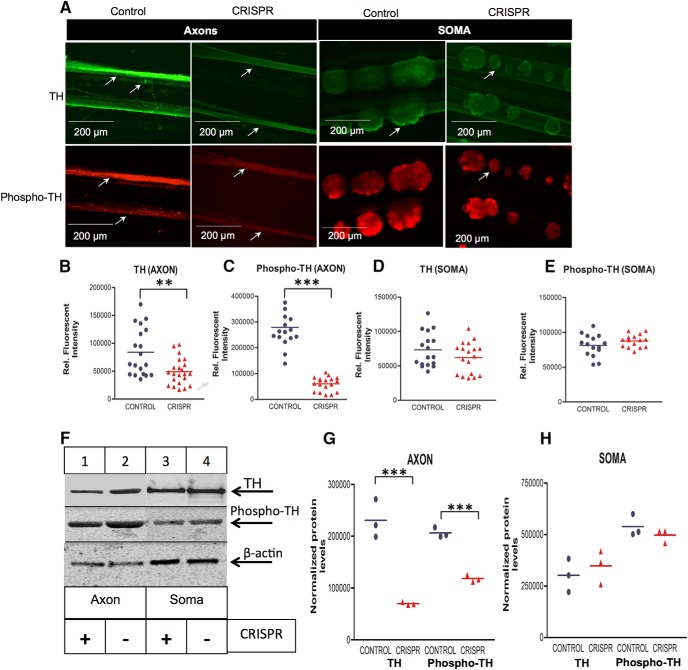
CRISPR-mediated deletion of the zip-code in the 3’UTR of TH mRNA selectively reduces axonal levels of TH protein and TH phosphorylation at SER40 (phospho-TH) in SCG neurons. ***A***, Intraaxonal TH and phospho-TH levels were measured using immunocytochemistry in axons and soma of SCG neurons infected with lentiCRISPR (control) or sgRNA 1 and sgRNA 2 containing lentiCRISPR (CRISPR). Decreased TH and phospho-TH levels are detected in axons of CRISPR neurons grown in Campenot chambers. Arrows denote axonal TH and phospho-TH. ***B–E***, Fluorescence intensity as a measure of TH (***B***, ***D***) and phospho-TH (***C***, ***E***) levels in axons and soma of SCG neurons were quantified using ImageJ, and fluorescence levels are provided as relative fluorescence intensity. Data are mean ± SEM from the measurement of 18–22 axons and 16–18 SCG ganglia from three independent experiments. Student’s *t* test, **p ≤ 0.002, ***p ≤ 0.0001. ***F***, Western blot analysis of axonal and cell body protein lysates from SCG neurons infected with lentiCRISPR (control) or sgRNA 1 and sgRNA 2 containing lentiCRISPR (CRISPR). β-actin was used as a loading control. ***G***, ***H***, TH and phopho-TH immunoblots of axonal or soma protein lysates of SCG neurons were quantified using ImageJ. Quantification showed significant reduction of TH and phospho-TH protein levels in axons one week after viral infection of SCG neurons grown in Campenot chambers, whereas TH and phospho-TH levels in parental cell soma remained unchanged. TH and phospho-TH band intensities were normalized to the protein levels detected for β-actin. Student's *t* test, ***p ≤ 0.0001.

To assess whether the CRISPR-mediated inhibition of axonal TH mRNA trafficking also resulted in reduced local synthesis and phosphorylation of TH, immunocytochemistry was employed to examine the levels of TH protein. Additionally, the phosphorylation of TH at Ser40 (phospho-TH) was monitored, since previous findings suggested that Ser40 phosphorylation is indicative of increased TH activity (Bevilaqua et al., 2001; Bobrovskaya et al., 2004). Both TH and phospho-TH levels were decreased by in the distal axons of CRISPR-infected neurons as compared with control neurons (TH: mean 84,000 ± 9780 control vs 49,240 ± 5030 CRISPR relative fluorescent intensity, *p* ≤ 0.002; phospho-TH: mean 278,800 ± 21,800 control vs 60,280 ± 6463 CRISPR relative fluorescent intensity, *p* ≤ 0.0001; Fig. 3*A–C*). Similar to the observation made for TH mRNA, while a decrease in TH and phospho-TH protein levels were detected in the distal axons of CRISPR-infected neurons, TH and phosphor-TH protein levels in the parental soma were little altered (TH: mean 73,290 ± 6047 control vs 62,210 ± 5314 CRISPR relative fluorescent intensity, *p* ≤ 0.17; phospho-TH: mean 81,240 ± 4067 control vs 87,780 ± 2377 CRISPR relative fluorescent intensity, *p* ≤ 0.18; Fig. 3*A*,*D*,*E*). Consistent with the immunocytochemistry findings, Western blot analysis of TH and phospho-TH in axonal and parental cell soma 7 d after viral infection showed reduced axonal levels of TH and phospho-TH, whereas their levels were unchanged in the parental soma [TH (axon): mean 230,700 ± 21420 control vs 69,980 ± 1669 CRISPR normalized protein levels, *p* ≤ 0.002; phospho-TH (axon): mean 206,300 ± 5487 control vs 118,400 ± 3862 CRISPR normalized protein levels, *p* ≤ 0.0002; TH (soma): mean 302,300 ± 46,380 control vs 347,500 ± 47,190 CRISPR normalized protein levels, *p* ≤ 0.53; phospho-TH (soma): mean 538,500 ± 30,640 control vs 497,300 ± 19,300 CRISPR normalized protein levels, *p* ≤ 0.32; Fig. 3*F–H* ]. These findings indicate that axonal trafficking of TH mRNA is critical for its local translation and activity in distal axons and nerve endings.

### Impaired axonal transport and local translation of TH mRNA reduces local catecholamine neurotransmitter levels

**Figure 4.**
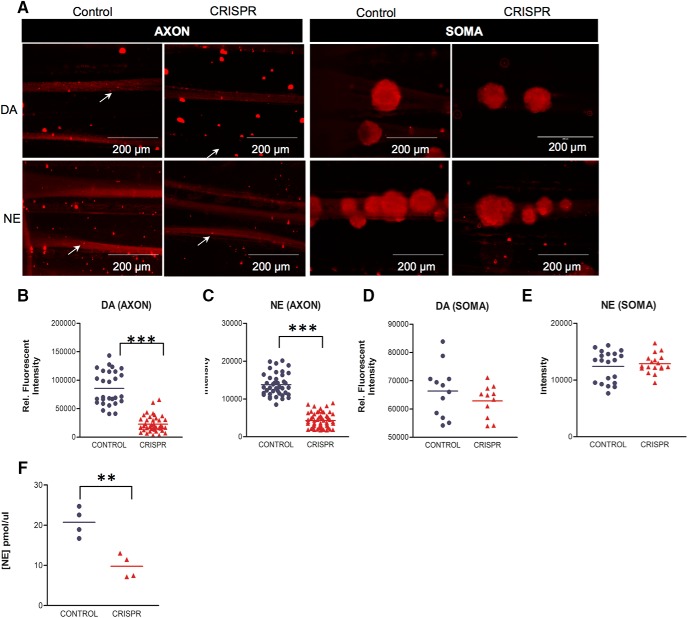
Reduced axonal transport of TH mRNA decreases axonal catecholamine levels in SCG axons. ***A***, Intra-axonal DA and NE levels were measured using immunocytochemistry in axons and soma of SCG neurons infected with with lentiCRISPR (control) or sgRNA 1 and sgRNA 2 containing lentiCRSPR (CRISPR). Arrows denote axonal DA and NE. ***B–H***, Fluorescence intensity as a measure of DA (***B***, ***D***) and NE (***C***, ***E***) levels was quantified in the soma and distal axons of SCG neurons, respectively, using ImageJ, and fluorescence levels are indicated as relative fluorescence intensity. Reduced DA and NE levels are detected in distal axons of CRISPR-treated neurons, whereas the parental soma DA and NE levels remained at control levels. Data are the mean ± SEM from the measurement of neurons cultured in six Campenot chambers from three independent experiments. ***F***, Distal axons located in the lateral compartment of Campenot chambers were treated for 10 min with 100 mM KCl. NE release into culture media was subsequently measured using an ELISA immunoassay, and NE concentration in the culture media was calculated using linear regression analysis. Reduced NE levels are detected in distal axons of CRISPR neurons, as compared with NE levels measured in the axons of control neurons. Values are mean ± SEM of four different experiments. Student’s *t* test, **p ≤ 0.003.

Since TH is the rate-limiting enzyme in catecholamine biosynthesis, we examined whether CRISPR-mediated ablation of axonal mRNA trafficking and local synthesis of TH affects catecholamine synthesis. Toward this end, SCG neurons cultured in Campenot chambers were infected with CRISPR or control viral particles, and 7 d after infection DA and NE levels were monitored using immunocytochemistry. DA and NE levels decreased by ∼70% in distal axonal compartments when compared with their levels in the axons of control neurons (DA: mean 84,100 ± 5840 control vs 26,310 ± 2173 CRISPR relative fluorescent intensity, *p* ≤ 0.0001; NE: mean 13,680 ± 512.4 control vs 4606 ± 243.0 CRISPR relative fluorescent intensity; *p* ≤ 0.0001; Fig. 4*A*–*C*). The measurement of DA and NE levels in the soma of CRISPR and control-infected neurons suggested that the reduction of catecholamine is restricted to the distal axons of SCG neurons, as no differences in the levels of soma DA and NE could be detected (DA: mean 66,370 ± 2670 control vs 62,860 ± 1715 CRISPR relative fluorescent intensity, *p* ≤ 0.29; NE: mean 12,410 ± 613.3 control vs 12,860 ± 388.8 CRISPR relative fluorescent intensity, *p* ≤ 0.54; NE: mean 13,680 ± 512.4 control vs 14,606 ± 243.0 CRISPR relative fluorescent intensity; *p* ≤ 0.52; Fig. 4*A*,*D*,*E*). Additionally, CRISPR-mediated inhibition of axonal TH mRNA trafficking and local synthesis resulted in an ∼50% reduction in NE release from the nerve endings of sympathetic neurons as assessed by an immunoassay (mean 20.73 ± 1.797 control vs 9.783 ± 1.464 CRISPR, *p* ≤ 0.003; Fig. 4*F*). These data provide evidence that the axonal trafficking and local translation of TH mRNA is contributing significantly to the synthesis of catecholamines in the axons of sympathetic neurons.

### Overexpression of the synthesis of TH mRNA in distal axons enhances local NE synthesis

**Figure 5.**
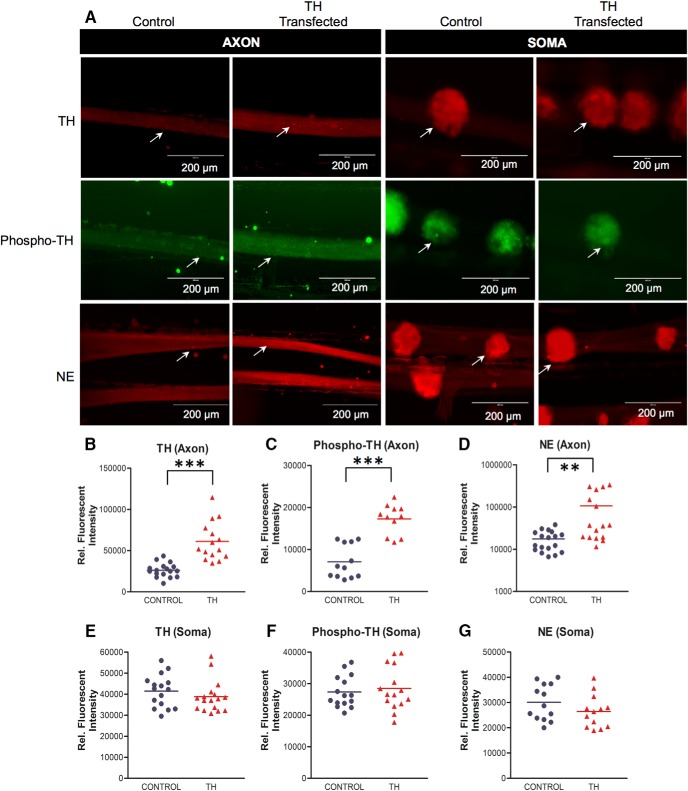
Local translation of TH mRNA enhances axonal levels of TH protein, TH phosphorylation at Ser40, and facilitates NE synthesis. ***A***, Intra-axonal TH, phospho-TH, and NE levels were measured 2 d after transfection using immunocytochemistry in axons lipofected with TH mRNA or sham-transfected controls. Increased levels of TH, phospho-TH and NE are detected in axons locally overexpressing TH, whereas the soma levels of TH, phospho-TH and NE remained at control levels. Arrows, denote axonal TH, phospho-TH and NE. ***B–D***, Fluorescence intensity as a measure of TH (***B***), phospho-TH (***C***), and NE (***D***) levels was quantified in the axons of SCG neurons using ImageJ, and fluorescence levels are provided as relative fluorescence intensity. Data are mean ± SEM from the measurement of 16–22 axons from three independent experiments. Student’s *t* test, **p ≤ 0.004; ***p ≤ 0.0001. ***E–G***, Fluorescence intensity as a measure of TH (***B***), phospho-TH, and NE (***D***) levels was quantified in the soma of SCG neurons using ImageJ, and fluorescence levels are provided as relative fluorescence intensity. Data are the mean ± SEM from the measurement of 16–22 SCG ganglia from three independent experiments.

CRISPR-mediated genome editing of the TH mRNA zip-code abolished axonal TH mRNA abundance and reduced its local translational activity. To further assess this finding, we examined whether the local overexpression of TH augmented cathecholamine synthesis. Toward this end, TH mRNA was transfected into the distal axons of SCG neurons and levels of TH and phospho-TH, as well as NE levels were assessed using immunocytochemistry. Transfection with TH mRNA resulted in an ∼2.5-fold increase in axonal TH protein levels compared with the endogenous TH levels in sham-transfected neurons (mean 26,240 ± 2046 control vs 61,190 ± 6028 TH mRNA transfected, relative fluorescent intensity, *p* ≤ 0.0001; Fig. 5*A*,*B*). In addition, TH phosphorylation at Ser40 in distal axons increased 2.5-fold above control levels neurons (mean 7059 ± 1147 control vs 17,780 ± 812.4 TH mRNA transfected, relative fluorescent intensity, *p* ≤ 0.0001; Fig. 5 *A*,*C*). In contrast, no significant differences in TH protein levels and TH phosphorylation at Ser40 were detected in the parental cell soma of sympathetic neurons (TH: mean 41,440 ± 1922 control vs 38,780 ± 1828 TH mRNA transfected, relative fluorescent intensity, *p* ≤ 0.32; phospho-TH: mean 27,350 ± 1288 control vs 28,470 ± 1777 TH mRNA transfected, relative fluorescent intensity, *p* ≤ 0.61; Fig. 5*A*,*E*,*F*). These findings suggest that enhanced TH enzymatic activity is restricted to distal axons on local synthesis of TH. To examine whether increased local TH synthesis contributed to elevation of catecholamine levels, NE levels were monitored 24 h after transfecting the distal axons with TH mRNA. As shown in Figure 5*A*,*D*, NE levels increased 5.5-fold in distal axonal compartments when compared with NE levels in sham-transfected axons (mean 17,660 ± 2216 control vs 107,100 ± 30,860 TH mRNA transfected, relative fluorescent intensity, *p* ≤ 0.004). In contrast, NE levels remained unaltered in the parental cell soma (mean 30,100 ± 1982 control vs 26,430 ± 1795 TH mRNA transfected, relative fluorescent intensity, *p* ≤ 0.18; Fig. 5*A*,*G*).

**Figure 6.**
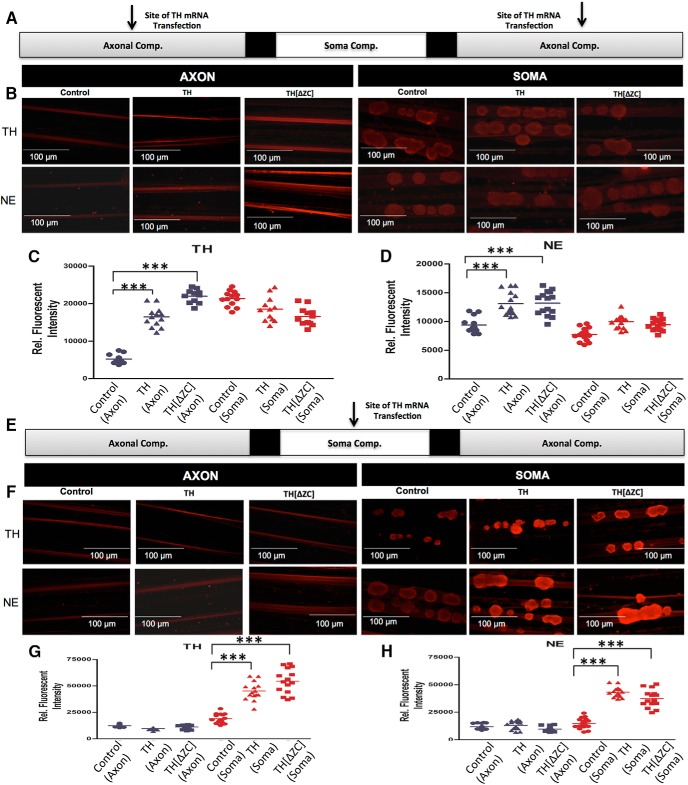
Intact and zip-code-less TH mRNAs are locally translated, enhance axonal levels of TH proteins and facilitate presynaptic NE synthesis. ***A***, Schematic representation of the Campenot culture chambers, the sites of mRNA transfection are indicated with arrows. ***B***, Intra-axonal and soma TH and NE levels were measured 1 d after transfection, using immunocytochemistry in soma and axons, in which axons were lipofected with intact or zip-code-less TH mRNA or sham transfected. Increased levels of TH and NE are detected in axons locally overexpressing TH, whereas the soma levels of TH, and NE remained at control levels. ***C***, ***D***, Fluorescence intensity as a measure of TH (***C***) and NE (***D***) was quantified in the axons and soma of SCG neurons using ImageJ. Fluorescence levels are provided as relative fluorescence intensity. Data are mean ± SEM from the measurement of 16–22 axons and 16–18 SCG ganglia from three independent experiments. One-way ANOVA, ***p ≤ 0.0001. ***E***, Schematic representation of the Campenot culture chambers, the site of mRNA transfection in the center compartment is indicated with an arrow. ***F***, Intra-axonal and soma TH and NE levels were measured 1 d after transfection, using immunocytochemistry in axons and cognate soma, in which soma were lipofected with intact or zip-code-less TH mRNA. Sham-transfected neurons served as negative controls. Increased levels of TH and NE are detected in soma and proximal axons locally overexpressing TH, whereas the distal axon levels of TH, and NE remained at control levels. ***G***, ***H***, Fluorescence intensity as a measure of TH (***G***) and NE (***H***) levels was quantified in the axons and soma of SCG neurons using ImageJ, and fluorescence levels are provided as relative fluorescence intensity. Data are the mean ± SEM from the measurement of 16–22 axons and 16–18 SCG ganglia from three independent experiments. One-way ANOVA, ***p ≤ 0.0001.

To extend the investigation of the local TH synthesis and activity, and to examine whether the axonal zip-code element is required for local TH protein synthesis, the full-length TH mRNA or a zip-code-less TH (TH[ΔZC]) mRNA were transfected into the distal axons of SCG neurons and the levels of TH and NE were measured in the axon and soma using immunocytochemistry. Transfection of axons with the intact or zip-code-truncated TH mRNAs resulted in significant elevation of the TH protein levels in the axons of SCG neurons 24 h after mRNA lipofection (mean 5213.54 ± 320.03 control vs 15,240.13 ± 1027 TH mRNA transfected, 27,302.87 ± 1306.7 TH[ΔZC] mRNAs transfected, relative fluorescent intensity, *p* ≤ 0.0001; Fig. 6*B*,*C*). In contrast, no significant differences in TH protein levels were detected in the parental cell soma of axons lipofected with full-length or zip-code-less TH mRNAs (mean 21,966.00 ± 657.74 control vs 19,505.47 ± 919 TH mRNA transfected, 17,748.53 ± 715.77 TH[ΔZC] mRNAs transfected, relative fluorescent intensity, *p* ≤ 0.0001). Consistent with the previous observation that local synthesis of TH results in enhanced NE levels, transfection of axons with the truncated TH mRNA also resulted in elevated axonal NE levels (mean 9901.33 ± 486.88 control vs 133,099.93 ± 524.67 TH mRNA transfected, 13,167.33 ± 537.20 TH[ΔZC] mRNAs transfected, relative fluorescent intensity, *p* ≤ 0.0001), whereas soma NE levels were marginally elevated, albeit not significantly, as compared with control levels (mean 8731.20 ± 292.11 control vs 11,210.87 ± 627.17 TH mRNA transfected, 9431.6 ± 257.83 TH[ΔZC] mRNAs transfected, relative fluorescent intensity; Fig. 6*B*,*D*). To examine whether intact or truncated TH mRNA can be locally translated in the soma and proximal axons, the center compartment of the Campenot chambers harboring the soma and proximal axons of SCG neurons were transfected with the full-length or zip-code-less TH mRNA. One day after transfection, pronounced levels of TH protein and NE were measured in the soma and proximal axons of SCG neurons, whereas the levels of TH and NE remained unchanged in the distal axons [TH (soma): mean 19,058 ± 1107.95 control vs 45,150.8 ± 2222.46 TH mRNA transfected, 54,456.73 ± 3015.53 TH[ΔZC] mRNAs transfected, relative fluorescent intensity, *p* ≤ 0.0001; TH (axon): mean 12,375.73 ± 350.21 control vs 10,672.67 ± 262.67 TH mRNA transfected, 11,158.94 ± 468.76 TH[ΔZC] mRNAs transfected, relative fluorescent intensity; NE (soma): mean 14,765.95 ± 983.07 control vs 43,148.67 ± 1222.14 TH mRNA transfected, 37,420.87 ± 2079.95 TH[ΔZC] mRNAs transfected, relative fluorescent intensity, *p* ≤ 0.0001; NE (axon): mean 11,790.4 ± 682.44 control vs 13,085.6 ± 1005.97 TH mRNA transfected, 10,661 ± 577.89 TH[ΔZC] mRNAs transfected, relative fluorescent intensity; Fig. 6*F–H* ].

Taken together, our results suggest that both inhibition and augmentation of local TH synthesis bidirectionally modulates axonal TH activity and catecholamine synthesis in cultured primary sympathetic neurons.

## Discussion

Previous studies demonstrated that TH is locally synthesized in axons, and that the axonal trafficking of TH mRNA is directed by a *cis*-acting regulatory element located in the 3’UTR of the transcript (Gervasi et al., 2016). In this report, evidence is provided to indicate that the zip-code plays a critical role in axonal TH mRNA trafficking and local TH synthesis in primary rat sympathetic neurons. Using CRISPR/Cas9-mediated gene editing, the axonal trafficking sequences located in the 3’UTR of TH mRNA were deleted. Genomic removal of this *cis*-acting regulatory element resulted in diminished axonal TH mRNA transport and local synthesis of TH. In this regard, it is postulated that this axonal localization signal functions as a nucleation site for the formation of ribonucleoprotein (RNP) complexes. These complexes are likely formed shortly after transcription and regulate the messages’ translation in the distal axonal compartment (Eom et al., 2003). Indeed, the results derived from *in situ* hybridization experiments suggest the presence of TH mRNA containing RNP puncta in distal axons of SCG neurons, which disappear on the genomic deletion of the zip-code: these findings suggest an essential role for the zip-code in the assembly of TH mRNA containing RNPs. At this time, one can only speculate on the composition of the RBPs, which bind to TH mRNA. Future studies employing RNA affinity pull-down-coupled mass spectrometry are likely to identify the RBPs which are enriched in the TH mRNA binding complex, as well as regulatory proteins that modulate the local translation of TH mRNA. In addition, RNA affinity purification, Western blot analyses, and siRNA-mediated knockdown experiments may prove useful in demonstrating that the TH zip-code indeed interacts with many trans-acting RBPs and that the expression of these proteins can modulate the transport of the TH transcript to the axon.

The control of the synthesis of catecholamine neurotransmitters is of fundamental importance, since changes in DA metabolism are associated with many neurologic disorders (Weinberger, 1987). The outcome of this study suggested that CRISPR-mediated deletion of the zip-code reduced axonal TH levels, TH phosphorylation at SER40, as well as diminished the axonal levels and release of NE. These findings suggest that the synthesis of the catecholamine neurotransmitters could be regulated locally in the terminal fields situated far distant from the parental cell soma. Interestingly, previous studies indicated that TH gene transcription and enzyme activation is sensitive to fast membrane potential changes and intracellular Ca^2+^-transients, that is, those associated with normal rates and patterns of neuronal activity (Aumann and Horne, 2012). More recently, a number of studies have also provided support for an activity-dependent regulation of axonal synthesis of proteins that play a critical role in the functioning of the presynaptic nerve terminals (Antar et al., 2006; Gomes et al., 2014). Although currently there is no evidence for an activity-dependent role for local TH synthesis, a recent post-mortem study assessing anomalies of the dopaminergic system in schizophrenia showed that TH protein levels were abnormal in schizophrenia, while mRNA expression levels were not affected, indicating that TH pathology in the substantia nigra/ventral tegmental area may occur post-transcriptionally (Perez-Costas et al., 2012). Interestingly, the administration of reserpine, a powerful catecholamine depleting agent, markedly enhanced TH mRNA levels in the cerebellum, a finding that raised the possibility that the local synthesis of TH might function to facilitate the restoration of neurotransmitter levels (Melia et al., 1994). In addition, in midbrain dopaminergic neurons, TH synthesis can be regulated at the translational level and synthesis of the enzyme can be induced in a cyclic AMP-dependent manner in the absence of alteration in mRNA levels (Chen et al., 2008). Taken together, these findings, along with the results shown in this report, raise the intriguing possibility that the synthesis of the catecholamine neurotransmitters could be regulated locally in the presynaptic nerve terminal.

Previous research suggested a neuromodulatory role for Angiotensin II (Ang II) on TH and DA-β-hydroxylase (DβH) actions, in part, by influencing the transcription of their genes (Gelband et al., 1998). The diverse physiologic effects of Ang II on catecholamine synthesis are mediated by its interaction with the angiotensin type 1 receptor subtype (AT_1_ receptor) coupled with the MAP kinase signaling pathway, which regulates enhanced neuromodulatory actions of Ang II including stimulation of TH, DβH, and NE transporter (Duff et al., 1995). Interestingly, the MAPK family includes signal-transducing enzymes that are involved in many aspects of cellular responses, including cue-mediated local protein synthesis in distal axons and dendrites. The activation of translation initiation regulators (i.e., phosphorylation of eIF-4E, eIF-4EBP1, and Mnk-1) in growth cones is dependent on p42/p44 and p38 MAPK (Campbell and Holt, 2003; Kelleher et al., 2004). Moreover, the local synthesis of nascent TH protein in distal axons may provide a critical entity for enzyme activation through a series of phosphorylation events mediated by upstream signaling, including ANG II-induced signaling, at the site of neurotransmitter release. Given the nature of Ang II function to chronically exert diverse physiologic actions in both peripheral and control neural tissues by activating the catecholamine synthetic machinery, it is tempting to speculate that ANG II regulation of local translation may result in the local synthesis of TH in the distal axons.

At present, whether mRNAs encoding the biosynthetic enzymes regulating the synthesis of other amino acid-derived neurotransmitters is unknown. However, mRNAs coding for the neuropeptides oxytocin, vasopressin and dynorphin are transported to the distal axons of peptidergic neurons comprising the hypothalamo-hypophyseal tract (Mohr et al., 1991). Hence, it is possible that mRNAs coding for a wide variety of neurotransmitters and/or neuromodulatory substances can be functioning locally in the axon and presynaptic nerve terminal.

In conclusion, the outcome of this study demonstrates that the local synthesis of TH can modulate the levels of catecholamines in the axon and nerve terminal, and raises the possibility that trafficking and local synthesis of TH mRNA may act to facilitate the restoration of neurotransmitter levels under conditions of prolonged release.

## Synthesis

Reviewing Editor: Vivian Budnik, University of Massachusetts Decisions are customarily a result of the Reviewing Editor and the peer reviewers coming together and discussing their recommendations until a consensus is reached. When revisions are invited, a fact-based synthesis statement explaining their decision and outlining what is needed to prepare a revision will be listed below. The following reviewer(s) agreed to reveal their identity: John Houle, François Giudicelli.

Summary of review

There is uniform agreement that the manuscript addresses a significant and timely question and that the results enhance our understanding of local translation as a regulatory mechanism potentially controlling neurotransmission. There are, however, a few issues that must be addressed before the manuscript is deemed suitable for publication at eNeuro.

A. Experimental:

1. Conclusions derived from Figure 5 require additional data: a comparison between transfection in axons vs soma of (1) intact TH mRNA and (2) zipcode-less TH mRNA. If the author's hypothesis is correct, zipcode-less mRNA should promote TH synthesis/activity in axons only when directly transfected into axons.

B. Quantifications:

1. Show quantification of NE levels in parental soma

2. quantify TH and phospho-TH in Western blots

3. quantify TH granules in axons as a function of axon length units

4. measure the proportion of intact vs indel TH by qRT-PCR

C. Corrections to text and figures:

1. Correct figure presentation and labeling, figure citations, and figure legends according to reviewer1 and 2 comments (appended)

2. verify fixation method, and state PCR primers used in the Methods section

3. correct the description of Fig.1B in the text (see reviewer's comments)

Reviewer's comments

Reviewer 1

This is an interesting and important study that extends our understanding of the role of mRNA transport and intra-axonal translation for local regulation in the axon and/or presynaptic terminal. The in vitro model is well established and the results clearly lay out the ability to modulate protein levels within axons as a result of altering mRNA trafficking. A critical feature is the progression of the study, from detecting decreased mRNA levels to reduced enzyme and enzyme phosphorylation levels and finally to reduced levels of the protein product (neurotransmitter). In the case of norepinephrine there also was decreased release from axon terminals.

There are some minor questions about the potential in vivo application of this work and some of the data presented in the text is not included in the indicated Figure (Figure 5). Some questions about methods also are raised.

1) There should be some discussion about the practical nature of the ability to regulate mRNA trafficking in general and more specifically about how one might manipulate the system to result in an increase in trafficking to affect local protein levels. In this study the authors demonstrate the ability to decrease neurotransmitter levels but this would not always be the desired outcome for targeted mRNAs and their protein product. The last sentence of the Discussion hints at this possibility but it is not clear how the present study indicates the potential to manipulate (increase) neurotransmitter levels after a prolonged release.

2) Please check that lanes in Figure 1 are labeled correctly. It is not clear why there would be decreased TH 3'UR and 44% indel in the Control. Should the legend for Figure 1 line 569 read “slower” migrating band, not “lower”?

3) On page 16 (line 383) presentation of data regarding transfection of distal axons with TH mRNA indicates Figure 5E shows phosphor TH in parental soma, but there is no Figure 5E.

4) Line 387, do you mean Fig 5 A, D for NE levels?

5) Line 391, there is no Figure for quantification of NE levels in parental soma. Figure 5D is NE in axons.

6) Line 201, check formalin concentration used for ISH.

7) Line 339, please add quantitative data for Western blots to demonstrate significant reduction of axonal levels of TH and phosphor TH?

8) For the quantification of TH granules in axons, it would be more accurate to present this as number per unit length of axon.

Reviewer 2

Major comments:

-The authors show that approximately half of the TH loci in CRSPR neurons harbor the intended deletion (Fig1B). Therefore, the RT-PCRs in Fig2A measure a mixture of intact and indel TH mRNAs. It would bring much support to the authors' interpretation if they measured this proportion of intact vs indel TH mRNA. This could be done rather easily by performing the same PCR reaction as in Fig1B on the cDNAs used for Fig2A, instead of genomic DNA.

Without this data, it is difficult to properly interpret the results of Fig2 since we do not know whether the intact and indel forms of mRNA have similar abundance in the soma and whether what can be seen in the axons represents mainly residual intact mRNA, or indel mRNA that can still be transported.

Incidentally, lines 291-292 in the “Results” section do not describe Fig1B accurately enough. It should be specified: « deletion of the zipcode in 44% of the TH loci ». Note that this does not mean 44% of the cells, as the Legend says: it is quite likely that some cells have only one affected allele, while the other is not deleted (for example, because it has lost one of the Crspr sites after cut and imperfect repair).

-While the first 4 figures convincingly support a role for TH mRNA zipcode in axonal synthesis of TH and catecholamines, the last part of the results (Fig5) is still too preliminary. In order to really support the idea that axonal TH mRNA promotes local TH synthesis and activity, the authors would need at least to compare 4 conditions: intact TH mRNA transfected in axons vs soma, and zipcode-less TH mRNA transfected in axons vs soma. The prediction is that zipcode-less mRNA could promote TH synthesis/activity in axons only when it is directly brought to axons by transfection.

-The Western blot shown in Fig3D lacks quantification. The image fails to convince that there is an important decrease of (phospho)-TH in CRSPR axons. Quantification of the stripes would help.

Minor comments:

-In Fig2A and 4F, each bar represents the mean and sem values of only 3-4 numbers: this would be better presented in the form of univariate scatterplots, as advocated by “Weissgerber at al. Beyond bar and line graphs: time for a new data presentation paradigm. PLoS Biol. 2015;13: e1002128”. When the dataset is small, presenting the full data is a much better information than mean and sem values. Along the same lines, all the other bar graphs would greatly benefit from being presented as boxplots, which represent the distribution of data more completely and faithfully than a simple mean+sem.

-The Material and Methods section on RT-PCR should mention the sequence of the primers used for PCR, or a reference to a publication that uses the same primers. This is particularly interesting for TH mRNA.

-The presentation of Figure 1 could be greatly improved. The two strands are not properly aligned. The arrows representing the gRNA are not properly positioned. The 5' and 3' labels are not adequately positioned.
